# Machine Learning to Predict Lower Extremity Musculoskeletal Injury Risk in Student Athletes

**DOI:** 10.3389/fspor.2020.576655

**Published:** 2020-11-19

**Authors:** Maria Henriquez, Jacob Sumner, Mallory Faherty, Timothy Sell, Brinnae Bent

**Affiliations:** ^1^Department of Statistics, Duke University, Durham, NC, United States; ^2^Department of Biology, Duke University, Durham, NC, United States; ^3^Michael W. Krzyzewski Human Performance Laboratory (K-Lab), Duke University, Durham, NC, United States; ^4^Department of Biomedical Engineering, Duke University, Durham, NC, United States

**Keywords:** injury risk, machine learning, sports science, student athlete, random forest

## Abstract

Injury rates in student athletes are high and often unpredictable. Injury risk factors are not agreed upon and often not validated. Here, we present a random-forest machine learning methodology for identifying the most significant injury risk factors and develop a model of lower extremity musculoskeletal injury risk in student athletes with physical performance metrics spanning joint strength measured with force transducers, postural stability measured using a force plate, and flexibility, measured with a goniometer, combined with previous injury metrics and athlete demographics. We tested our model in a population of 122 student athletes with performance metrics for the lower extremity musculoskeletal system and achieved an injury risk accuracy of 79% and identified significant injury risk factors, that could be used to increase accuracy of injury risk assessments, implement timely interventions, and decrease the number of career-ending or chronic injuries among student athletes.

## Introduction

Musculoskeletal injuries are a significant problem in student athlete populations with some reports indicating that 90% of student athletes report a sports-related injury during their athletic career as a student (Research Report: Changing the Culture of Youth Sports, 2014). Student athletes are 2.5 times more likely to report a major injury and chronic injuries than non-athletes: 67% of former Division I athletes sustain a major injury and 50% reported chronic injuries in a survey of 232 former Division I athletes (Simon and Docherty, 2014; Cowee and Simon, 2019). These injuries will have chronic effects that may influence lifelong physical activity behavior (Hrysomallis, 2007) and have profound financial implications for student athletes including costly medical bills and potential loss of scholarship (Dixon, 2017). The lower extremity musculoskeletal system consists of 42% of all injuries obtained while engaging in sports and recreation activities (Conn et al., 2003). Training interventions can help mitigate lower extremity musculoskeletal injuries (Patel et al., 2017); however, the identification of risk factors has been challenging. There is a critical need to identify risk factors for lower extremity musculoskeletal injuries.

The identification of risk factors of lower extremity musculoskeletal injuries has been challenging because of the lack of consensus on risk factors. Proposed risk factors for lower extremity musculoskeletal injury risk include over-training, inadequate nutrition, previous injury, gender, limb dominance, ankle and knee joint laxity, muscle strength, imbalance, and postural stability (Neely, 1998; Murphy et al., 2003; Borresen and Lambertt, 2009; Saragiotto et al., 2014; Toohey et al., 2017). Machine learning models have previously been developed to explore risk factors in specific populations (Meeuwisse, 1994; Bahr and Holme, 2003; Novatchkov and Baca, 2013; Tixier et al., 2016; Bunker and Thabtah, 2019; Claudino et al., 2019). Relevant studies using machine learning to assess injury risk in sports include injury risk in elite youth football using gradient boosting algorithms with an accuracy of 85% (Rommers et al., 2020), injury risk in professional soccer players using multiple machine learning methods with an ROC score = 0.747 (López-Valenciano et al., 2018) and injury risk in the NBA using game day statistics and a sliding window with random forests, which achieved an AUC of 0.65 – 0.95 depending on the length of the sliding window (Talukder et al., 2016). However, previous machine learning models have been limited in their inclusion of measurements of muscle strength, imbalance, flexibility, and postural stability. With the inclusion of these physical performance measurements, machine learning models could be utilized to determine significant physical performance risk factors for injury risk, which would be advantageous to modeling and understanding injury risk factors.

Risk factors of lower extremity musculoskeletal injury risk need to be identified in order to implement timely interventions to reduce the prevalence of injury in student athletes. In this study, we will explore the use of machine learning algorithms to identify the most significant risk factors and develop a model of lower extremity musculoskeletal injury risk with physical performance measurements for the lower extremity musculoskeletal system in student athletes.

## Methods

### Participants

**Figure 1:**
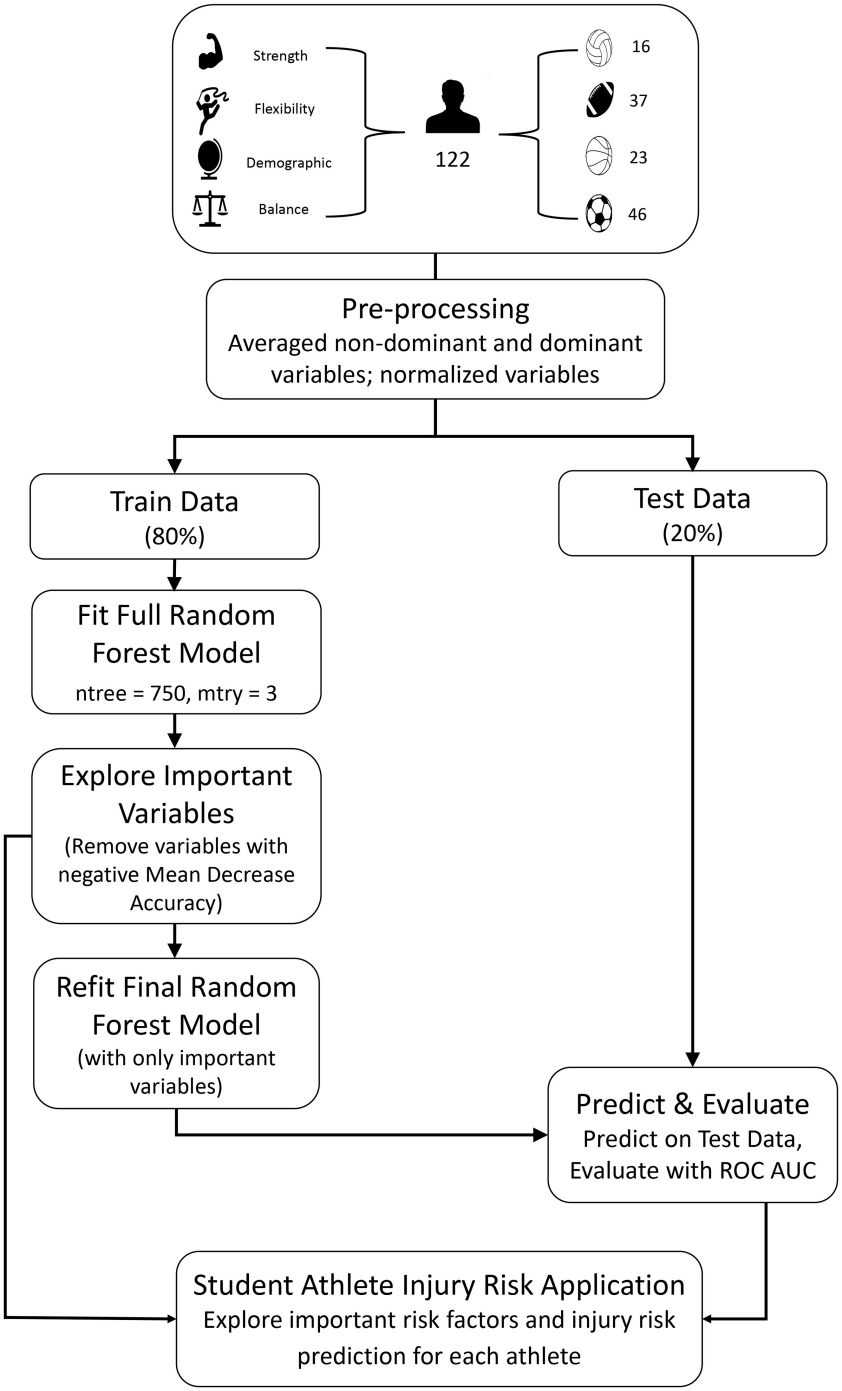
Graphical Abstract of study. Overview of dataset and data and machine learning pipelines.

The study population was composed of 122 college Division I NCAA athletes (51 females, 71 males) across four sports: men's and women's basketball (18.9%), men's football (30.3%), men's and women's soccer (37.7%), and women's volleyball (13.1%) (Table 1, Figure 1). The average age of the study population was 19.56 ± 1.33 years (Table 1). The percentage of student athletes in our data set with any occurrence of a lower extremity injury in the year following their initial testing date was approximately 43.44%. All participants gave written informed consent by a university institutional review board.

**Table 1:** Demographics.

	**Number of Participants**	**Percentage (%)**
**SPORT**		
Women's Basketball	12	9.38%
Men's Basketball	11	9.02%
Women's Soccer	23	18.85%
Men's Soccer	23	18.85%
Women's Volleyball	16	13.11%
Men's Football	37	30.33%
**GENDER**		
Male	71	58.20%
Female	51	41.80%
**AGE**		
18–20	66	54.10%
20–22	42	34.40%
22+	14	11.50%

*Student athlete primary collegiate sport, self-identified gender, and age range of participants in the dataset*.

### Overview of Data Collection

The data contains 50 physical metrics spanning strength, postural stability, and flexibility (dominant and non-dominant leg) combined with previous injury binary classification and demographic data (Supplementary Table 1).

The postural stability assessments are performance metrics that are tested using a force plate (Kistler 9286A, Amherst, NY) at a sampling frequency of 1,200 Hz and quantify balance under both dynamic and static conditions. Postural stability data was collected and processed according to Sell (2012). The strength assessments are performance metrics that are tested using force transducers (seated and handheld) (Lafayette Instrument Company, Lafayette, IN) and assess joint strength. The flexibility assessments included performance metrics that tested joint range of motion and the muscle-tendon assessed with a standard goniometer or digital inclinometer (Saunders Group, Chaska, MN). Strength and flexibility data were collected and processed according to Faherty et al. (2020). Additionally, binary classification of previous injury in the past 12 months, age, and gender were collected.

### Model Development and Evaluation

Prior to model development, features of dominant and non-dominant leg were averaged together and normalized using z-scores, as shown in Equation 1 (Altman, 1968). While normalization is not necessary for random forest modeling due to it being tree-based, we used the normalized features to get units in a comparable form in order to aid in understanding of feature differences for a web-based application for assessing injury risk in student athletes. The final feature set contained 2 categorical variables and 19 normalized numeric variables (Supplementary Table 1). The outcome variable was a binary classification of injury in the year following initial data collection. Statistical descriptions of the dataset can be found in our public repository: https://github.com/marhenriq/Student-Athlete-Injury-Risk.

(1) $$\begin{array}{l} normVariable=\;\frac{VALUE-mean\left(VALUE\right)}{std\left(VALUE\right)} \end{array}$$

In order to identify the most significant risk factors and develop a model of lower extremity musculoskeletal injury risk in student athletes, we required a machine learning model that could handle the breadth of highly-correlated physiological features and distributions in our dataset while also being interpretable to enable our differentiation of the most important risk factors. Random forest models are ideal for our use case because they have no formal distributional assumptions and are therefore useful in handling features with a variety of distributions (Ho, 1998; Kleinberg, 2000; Breiman, 2001). Random forest models can handle multi-modal data and they are interpretable, allowing us to decipher important relationships between features and outcome variables (Ho, 1998; Kleinberg, 2000; Breiman, 2001).

We developed a random forest (RF) model with the randomForest library in R that is based on Breiman's Random Forests (Breiman, 2001; Liaw, 2018) using a test/train split validation strategy (80:20 split, separating on subjects) and tuned hyperparameters using a GridSearch (Figure 1). Our RF model was composed of 750 trees (number of variables available for splitting at each tree node, mtry = 3). We tuned the RF model by removing all variables that yielded a negative Mean Decrease Accuracy score. Mean decrease accuracy (also known as permutation importance) is the scaled decrease in accuracy if the variable is removed from the features when developing the model (Han et al., 2016). The variables removed from the final model because they had a negative mean decrease accuracy were eyes closed balance test composite score, DPSI composite score, gender, gastrocnemius flexibility, and knee extension strength.

We set the output of our model to be probabilities of a participant being injured in the following year (Subject Injury Risk). We chose probabilities as the output of our model rather than using a scoring system with no maximum value because probabilities are more interpretable due to being bound from values 0 to 1. We used the confusion matrix to define the class error rates of false negative and false positive errors in our model. The performance metric we utilized to evaluate the performance of our model was receiver operating characteristic (ROC) area under the curve (AUC). We completed a secondary validation of our RF model using k-fold cross validation (folds = 5, mtry = 2). All data analyses and model development were performed in R (version 3.2.3).

### Web Application

To facilitate the communication of results with athletes, coaches, and physicians, we developed a web-application through Dash by Plotly^1^ that allows the viewer to see how respective individual statistics/testing values compare with that of the individual's team (Figure 1). Backend development of the application was coded in Python, while frontend development was coded in HTML and CSS. The application was deployed through Heroku (Salesforce, San Francisco, CA).

## Results

**Figure 2:**
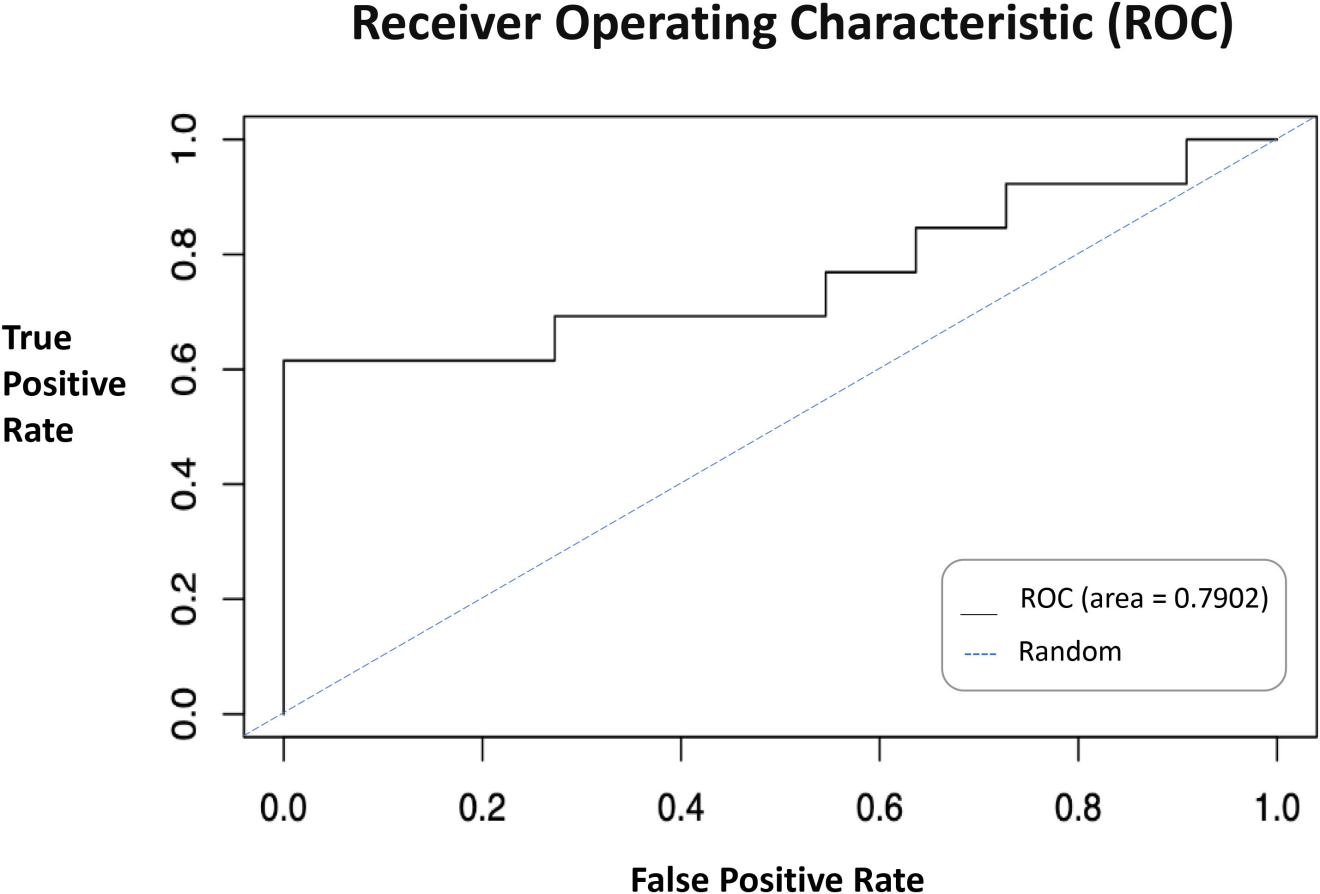
Random Forest ROC. ROC of test/train validated model. Black line shows results of the model presented in this study. Random chance is represented with the blue dashed line.

We developed a model for injury risk in student athletes using random forest machine learning. With our initial validation strategy of test/train split, our ROC AUC accuracy metric was 79.02% (Figure 2). The false negative class error rate was 15.52% and the false positive class error rate was 77.50%. So, errors in our model were more likely to be false positives than false negatives. Our secondary validation with k-fold cross validation resulted in an average ROC AUC of 68.90%. Comprehensive analysis of the random forest model is available in a public repository at https://github.com/marhenriq/Student-Athlete-Injury-Risk.

**Figure 3:**
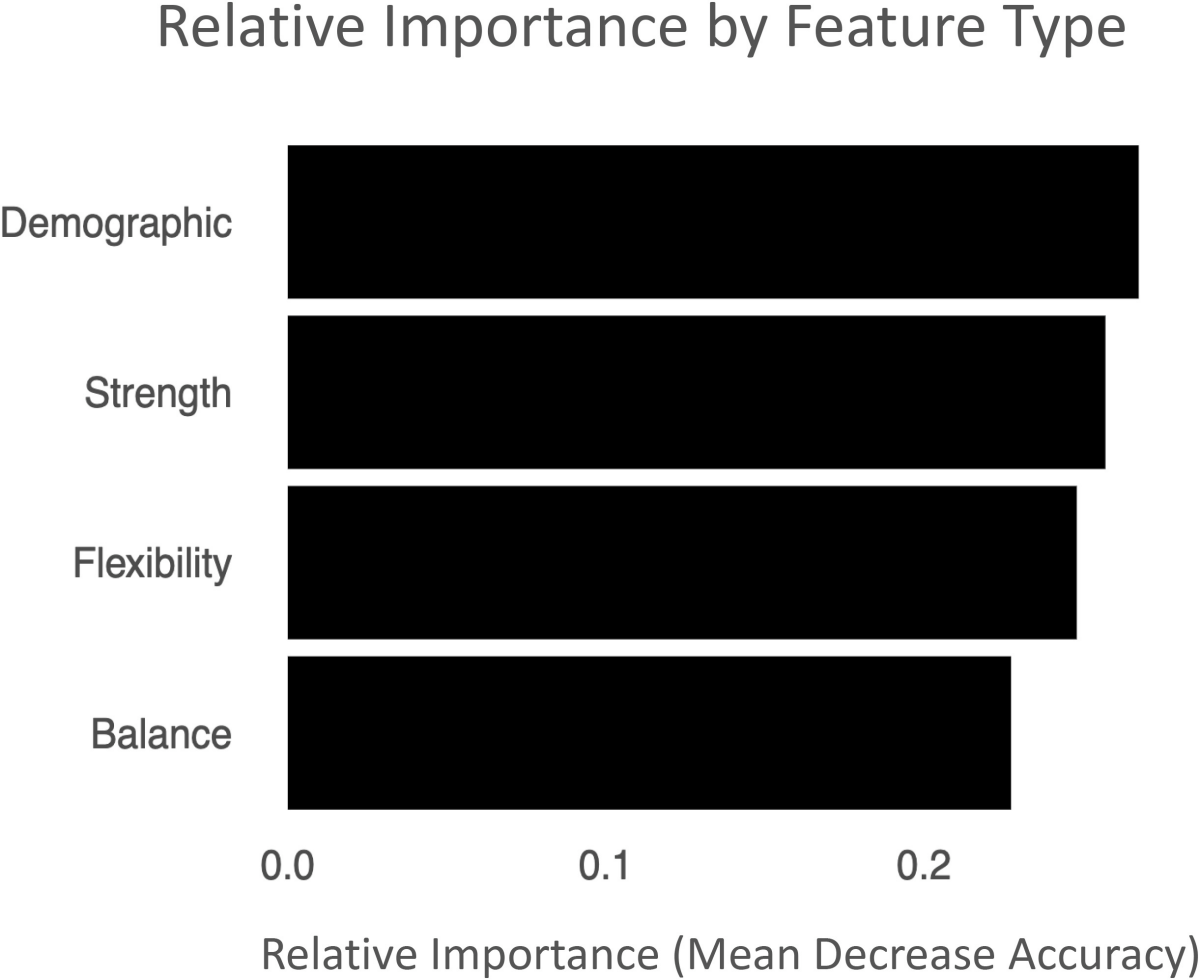
Relative Variable Importance by Feature Type. Importance of feature types in RF model using relative Mean Decrease Accuracy as the metric.

We determined the features most important in determining risk of injury in our model using mean decrease accuracy. The most important variables include Hip External Rotation Strength, Hip Adductor Strength, and Straight Leg Raise (Table 2, Supplementary Figure 1, Supplementary Table 2). We examined which feature type (strength, flexibility, balance, and demographics) were most important in determining risk of injury and found that all feature types were important in model development; the relative importance of each feature type were within 0.03 of each other (Figure 3).

**Table 2:** Random Forest Variable Importance.

**Variable**	**Category**	**Mean Decrease Accuracy**
Hip adductor strength	Strength	5.3465738
Hip external rotation strength	Strength	4.3168850
Straight leg raise	Flexibility	4.1723271
Height	Demographic	3.6593198
Hip abductor strength	Strength	3.6520573
Hip internal rotation strength	Strength	3.4898969
Eyes open balance test composite score	Balance	3.2547060
Ankle dorsiflexion strength	Strength	2.9879077
Ankle plantarflexion strength	Strength	2.8693613
Primary sport type	Demographic	2.7218000
Knee flexion strength	Strength	2.3598642
Eyes closed balance test composite score	Balance	2.1693963
Active knee extension	Flexibility	1.7146625
Ankle eversion strength	Strength	1.6240054
Ankle inversion strength	Strength	0.8680607

*Variable Importance represented by Mean Decrease Accuracy for 15 variables used in tuned model*.

The outputs of our model were used in an application for assessing injury risk in student athletes (Supplementary Figure 2). This application allows the comparison of personal injury risk to team injury risk, creating a normalized injury risk to the student athletic team. Additionally, this application enables the comparison of important features of injury risk among student athletes on the same team, which can be used to develop injury risk mitigation training programs for student athletes.

## Discussion

It is critical to identify risk factors of lower extremity musculoskeletal injury risk in order to implement timely interventions to reduce the prevalence of injury in student athletes. In this study, we utilized random forest machine learning algorithms to identify the most significant risk factors and develop a model of lower extremity musculoskeletal injury risk with physical performance measurements for the lower extremity musculoskeletal system in student athletes. Specifically, we developed a random forest machine learning model to explore risk factors of lower extremity musculoskeletal injury in student athletes. We used these important risk factors to create a model for student athlete injury risk. By combining analysis of identified risk factors and injury risk relative to the entire student athlete team, coaches, sports injury clinicians, and athletes can quantitatively summarize the strengths and weaknesses of an athlete, enabling the implementation of timely interventions to reduce student athlete injury risk.

This model and the web-based application is meant to enable athletes, coaches, and trainers to assess relative injury risk and provide guidance on what physical performance metrics need to be improved to decrease this risk.

On average, the relative importance values of each feature type (strength, flexibility, balance, and demographics) with respect to Mean Decrease Accuracy are quite similar (Figure 3, Table 2). This suggests that a combination of these areas is important to comprehensively assess student athlete injury, as supposed to one or two feature types. Removing one feature type entirely would significantly decrease the predictive power of the RF model. This model can serve as validation to utilize measurements across these feature types in injury risk assessment. The most important variables in our model of injury risk in student athletes were two hip strength-based metrics, Hip Adductor Strength and Hip External Rotation Strength, and a flexibility metric, Straight length Raise. These metrics were more important than ankle strength, knee strength, and balance metrics. This supports a body of literature that found relationships between hip strength and risk for injury (Holcomb et al., 2012; Khayambashi et al., 2016; Stastny et al., 2016; Mucha et al., 2017; Nguyen et al., 2017; Powers et al., 2017) and relationships between flexibility and risk for injury (Gleim and McHugh, 1997; Thacker et al., 2004; Leppänen et al., 2014). Surprisingly, ‘Primary Sport’ was not one of the most important variables in our model of injury risk. This could be due to our limited amount of sports and/or the small sample size in the dataset.

The large number of confounding variables present when recording injury is a significant obstacle in discovering the true accuracy of any injury risk model. A student athlete might have a very high risk for injury but can never get injured due to lack of playtime or successful training/physical therapy sessions and an athlete with a low injury risk could easily get injured through aggressive contact injury or unexpected confounding variables. Thus, it is nearly impossible to predict injury with a model. However, through the development of injury risk models, we can better understand the physiological parameters that have an impact on risk for injury. Accordingly, this study serves to normalize the significant physical performance metrics and demographic variables for the student athletes in order to quantitatively provide an overview of a student athlete's strengths and weaknesses. The risk score given by our model, normalized to teammates, should serve as a tool, that combined with insights from clinicians and coaches, can aid in the development of an injury-prevention plan to quantitatively lower an athlete's risk for injury. The use of any machine learning model for injury risk should be accompanied by additional visualizations and summary statistics of the student athlete as well as the student athlete's team to provide further insight into an athlete's risk for injury, as we have done in our student athlete injury risk application.

The limitations of this study include the small sample size (122 student athletes) and not having features addressing student athlete nutrition habits, stress factors, and game play statistics. While we have attempted to reduce training time and the potential for overfitting with careful feature selection methods, random forest modeling has inherent limitations, which include high model complexity requiring computational resources and longer training periods than other machine learning frameworks. We use Mean Decrease Accuracy for feature selection, which has been known to have limitations due to the multicollinearity problem (variable impact calculation is less accurate when there are high numbers of correlated variables) (Hur et al., 2017). Future studies with significantly larger sample sizes will be required to improve upon this framework for general student athlete injury risk. Different models for specific sports should be explored to determine specific injury risk triggers. While our model did not find Primary Sport to be a significant variable in injury risk, it should still be explored and validated. Models generated for specific types of lower extremity musculoskeletal injury may enable greater specificity and interpretability of injury risk. In future studies, gradient tree boosting, another tree-based machine learning model, could be considered to reduce the computational resources necessary for random forest modeling. Additionally, new methods of feature selection that solve the multicollinearity problem, such as the Shapley Value method (Hur et al., 2017), could be considered for future selection in future studies.

## Data Availability Statement

The datasets presented in this article are not readily available because of participant confidentiality and privacy restrictions. Requests to access the datasets should be directed to Brinnae Bent, brinnae.bent@duke.edu.

## Ethics Statement

The studies involving human participants were reviewed and approved by University of Pittsburgh Institutional Review Board. The patients/participants provided their written informed consent to participate in this study.

## Author Contributions

MH, JS, and BB were involved in data analysis and interpretation, model development, and application development. MF and TS were involved in study design and data collection. MH and BB were involved in manuscript preparation. MH, BB, TS, and MF were involved in manuscript editing. All authors contributed to the article and approved the submitted version.

## Conflict of Interest

The authors declare that the research was conducted in the absence of any commercial or financial relationships that could be construed as a potential conflict of interest.

## Footnotes

^1^*Dash Documentation and User Guide*. Plotly. Available online at: https://dash.plotly.com/ (accessed October 5, 2020).
