# Supplementary material for: Machine Learning to Predict Lower Extremity Musculoskeletal Injury Risk in Student Athletes
Source: Front Sports Act Living. 2020 Nov 19;2:576655. doi: 10.3389/fspor.2020.576655 (PMC7739722; doi:10.3389/fspor.2020.576655)

Supplementary Material

Supplementary Table 1. Explanation of Abbreviations. Definitions of the 21variable abbreviations from the full RF model. Please note that all definitions are normalized. Prior to normalization, the units of flexibility metrics are in degrees, strength variables are in percent body weight, and balance metrics are in ground reaction force (N).

**Abbreviation**	**Definition**
normHERS	Hip External Rotation Strength
normHABS	Hip Abductor Strength
normHIRS	Hip Internal Rotation Strength
normKFS	Knee Flexion Strength
normHADS	Hip Adductor Strength
normAES	Ankle Eversion Strength
normAIS	Ankle Inversion Strength
normKES	Knee Extension Strength
normAPF	Ankle Plantarflexion Strength
normSEC_COMPOSITE	Eyes Closed Balance Test Composite Score
normSEO_COMPOSITE	Eyes Open Balance Test Composite Score
normDPSI_COMPOSITE	DPSI Composite Score

normSLR	Straight Leg Raise
normAKE	Active Knee Extension
normADORSIS	Ankle Dorsiflexion Strength
normGAST	Gastrocnemius Flexibility
normWEIGHT	Weight
normHEIGHT	Height
GENDER	Gender
PRIMARY_SPORT_TYPE	Sport
normAGE	Age

Supplementary Table 2. Random Forest Variable Importance. Variable Importance represented by Mean Decrease Accuracy (no units) for all 21 variables (un-tuned model).

**Variable**	**Category**	**Mean Decrease Accuracy**
Hip External Rotation Strength	Strength	4.6136782
Hip Adductor Strength	Strength	3.5836804
Hip Internal Rotation Strength	Strength	3.3870181
Straight Leg Raise	Flexibility	2.6842210
Active Knee Extension	Flexibility	2.2702588

Sport	Demographic	2.1801716
Ankle Inversion Strength	Strength	1.5600919
Height	Demographic	1.4619164
Knee Flexion Strength	Strength	1.3444648
Ankle Eversion Strength	Strength	1.2905066
Ankle Plantarflexion Strength	Strength	1.1227158
Ankle Dorsiflexion Strength	Flexibility	1.0686056
Hip Abductor Strength	Strength	0.9626947
Weight	Demographic	0.8698267
Age	Demographic	0.7866845
Eyes Open Balance Test Composite Score	Balance	0.5482729
Eyes Closed Balance Test Composite Score	Balance	-0.3552219
DPSI Composite Score	Balance	-1.1455886
Gender	Demographic	-1.5249294
Gastrocnemius Flexibility	Flexibility	-1.9174240
Knee Extension Strength	Strength	-2.5810260

Supplementary Figure 1. Random Forest Variable Importance. Variable Importance by Mean Decrease Accuracy for 15 variables used in tuned model.

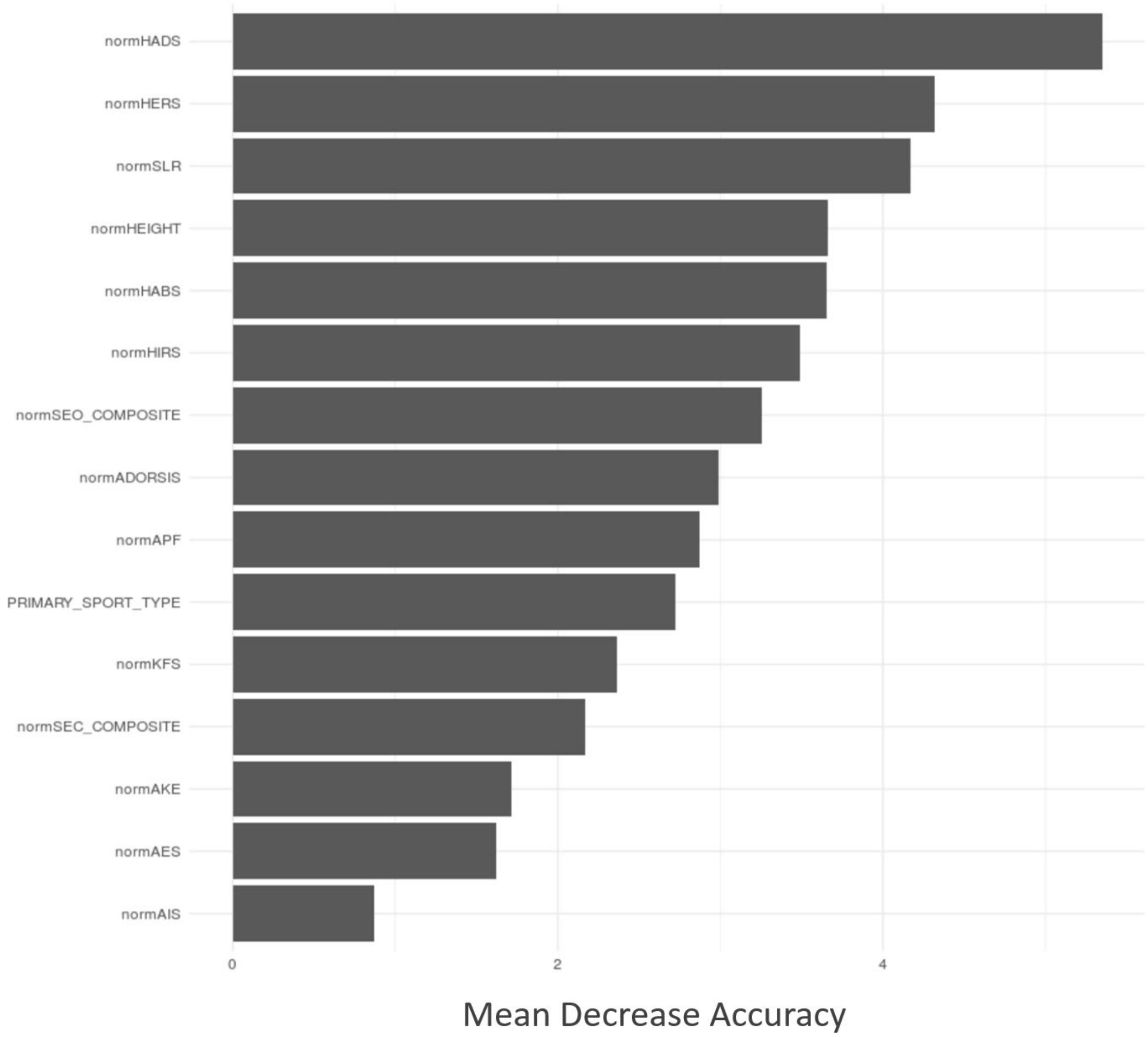

Supplementary Figure 2. Web Application for Student Athlete Individual Training Risk. The results from this model were incorporated in an extensive risk analysis for student athletes, coaches, and trainers to use in developing training tools to minimize injury risk.

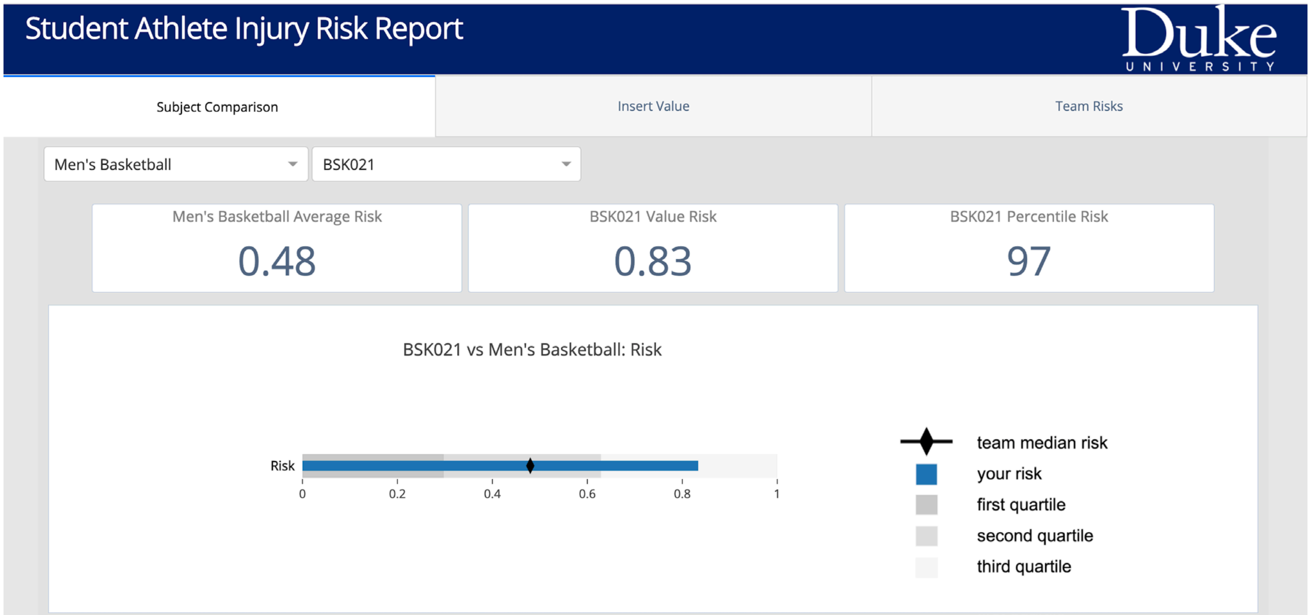

Supplement: Supplementary file 1 [file Presentation_1.PDF]
