# Supplementary material for: Machine Learning to Predict Lower Extremity Musculoskeletal Injury Risk in Student Athletes
Source: Front Sports Act Living. 2020 Nov 19;2:576655. doi: 10.3389/fspor.2020.576655 (PMC7739722; doi:10.3389/fspor.2020.576655)
Supplement: Supplementary file 2 [file Image_1.PDF]

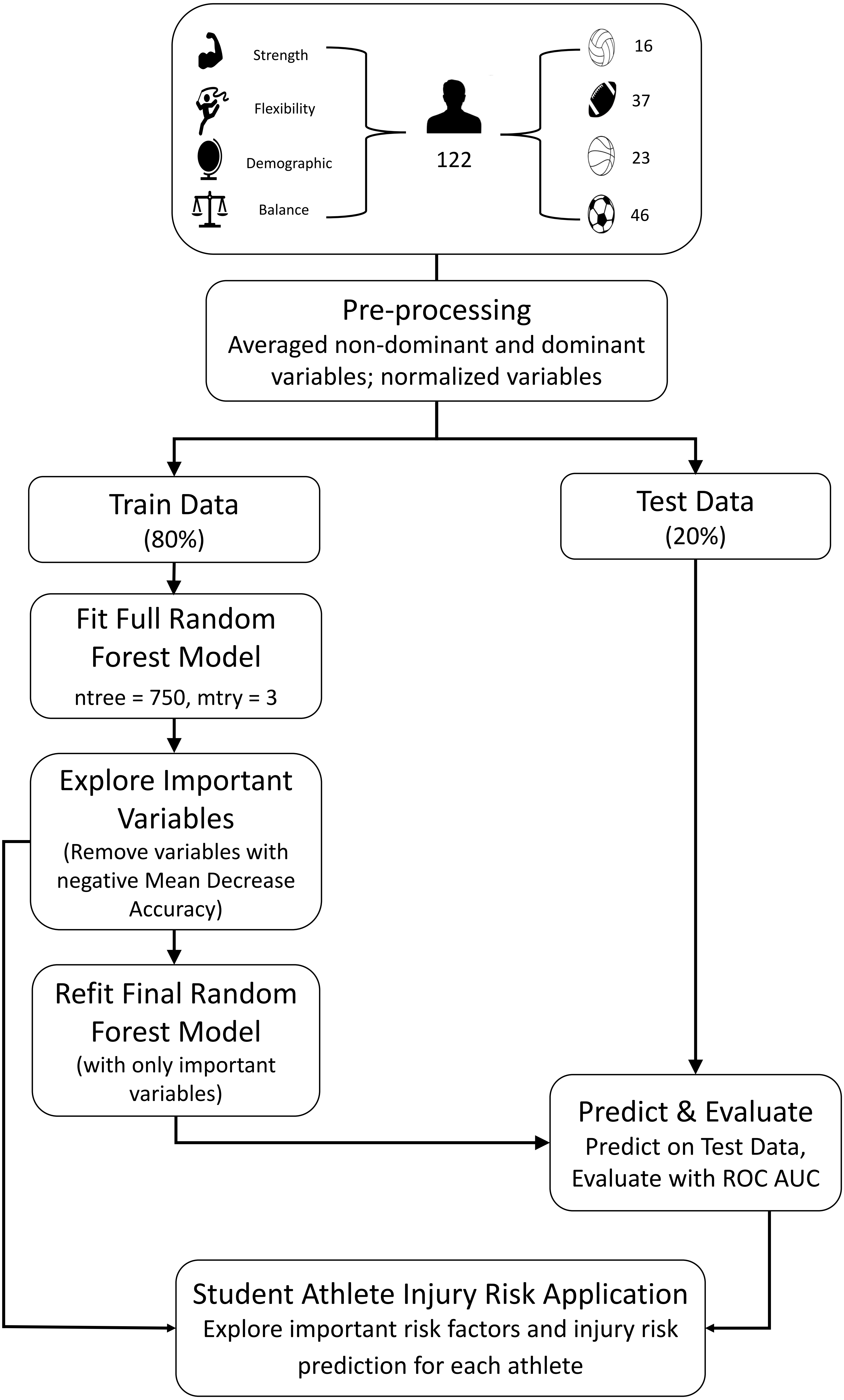
Strength
Flexibility
Demographic
Balance
122
16
37
23
46
Pre-processing
Averaged non-dominant and dominant variables; normalized variables
Train Data
(80%)
Test Data
(20%)
Fit Full Random Forest Model
ntree = 750, mtry = 3
Explore Important Variables
(Remove variables with negative Mean Decrease Accuracy)
Refit Final Random Forest Model
(with only important variables)
Predict & Evaluate
Predict on Test Data, Evaluate with ROC AUC
Student Athlete Injury Risk Application
Explore important risk factors and injury risk prediction for each athlete
