# Supplementary figures and images for: Machine Learning to Predict Lower Extremity Musculoskeletal Injury Risk in Student Athletes
Source: Front Sports Act Living. 2020 Nov 19;2:576655. doi: 10.3389/fspor.2020.576655 (PMC7739722; doi:10.3389/fspor.2020.576655)

# Relative Importance by Feature Type

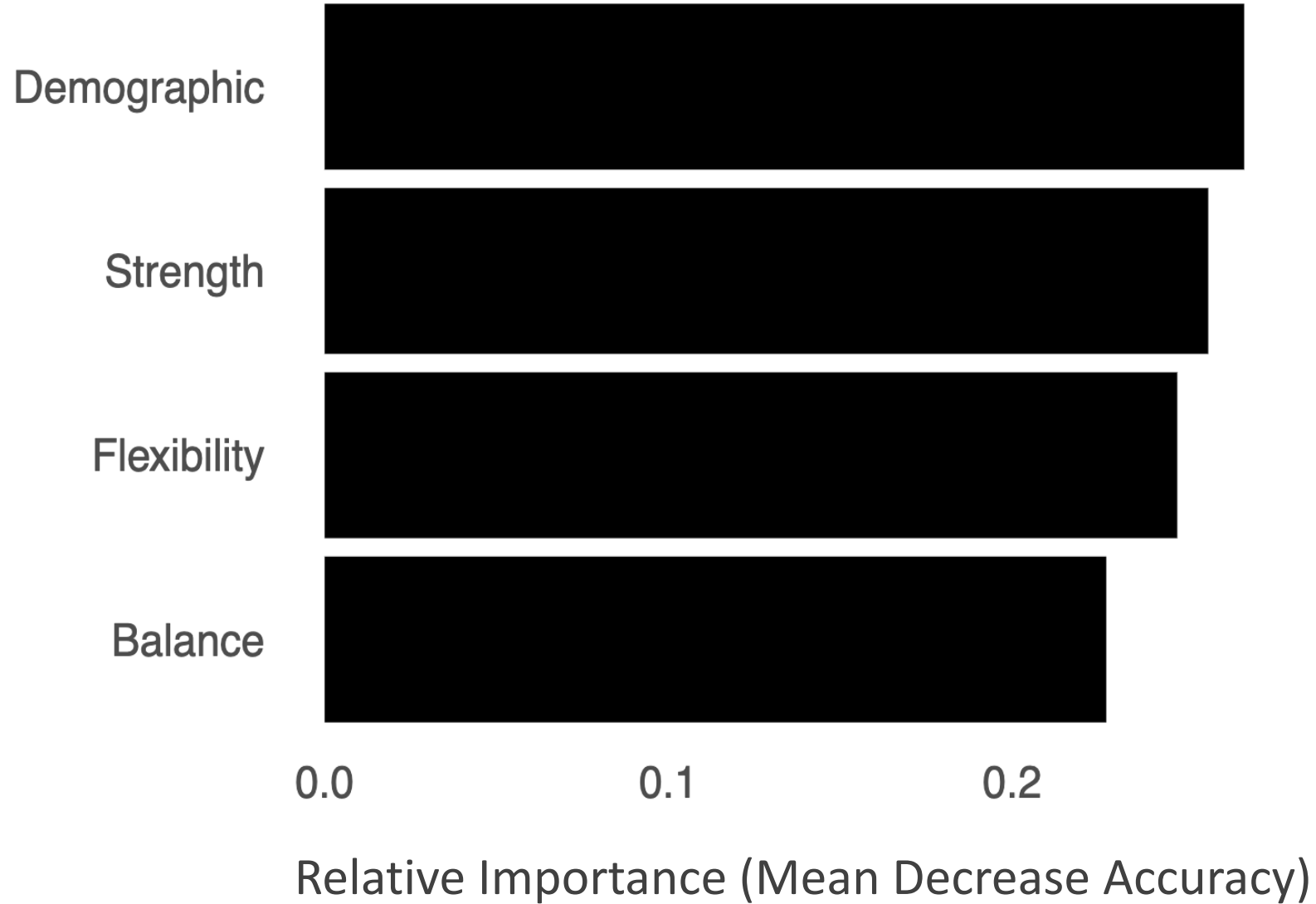

Supplement: Supplementary file 3 [file Image_2.PDF]

# Receiver Operating Characteristic (ROC)

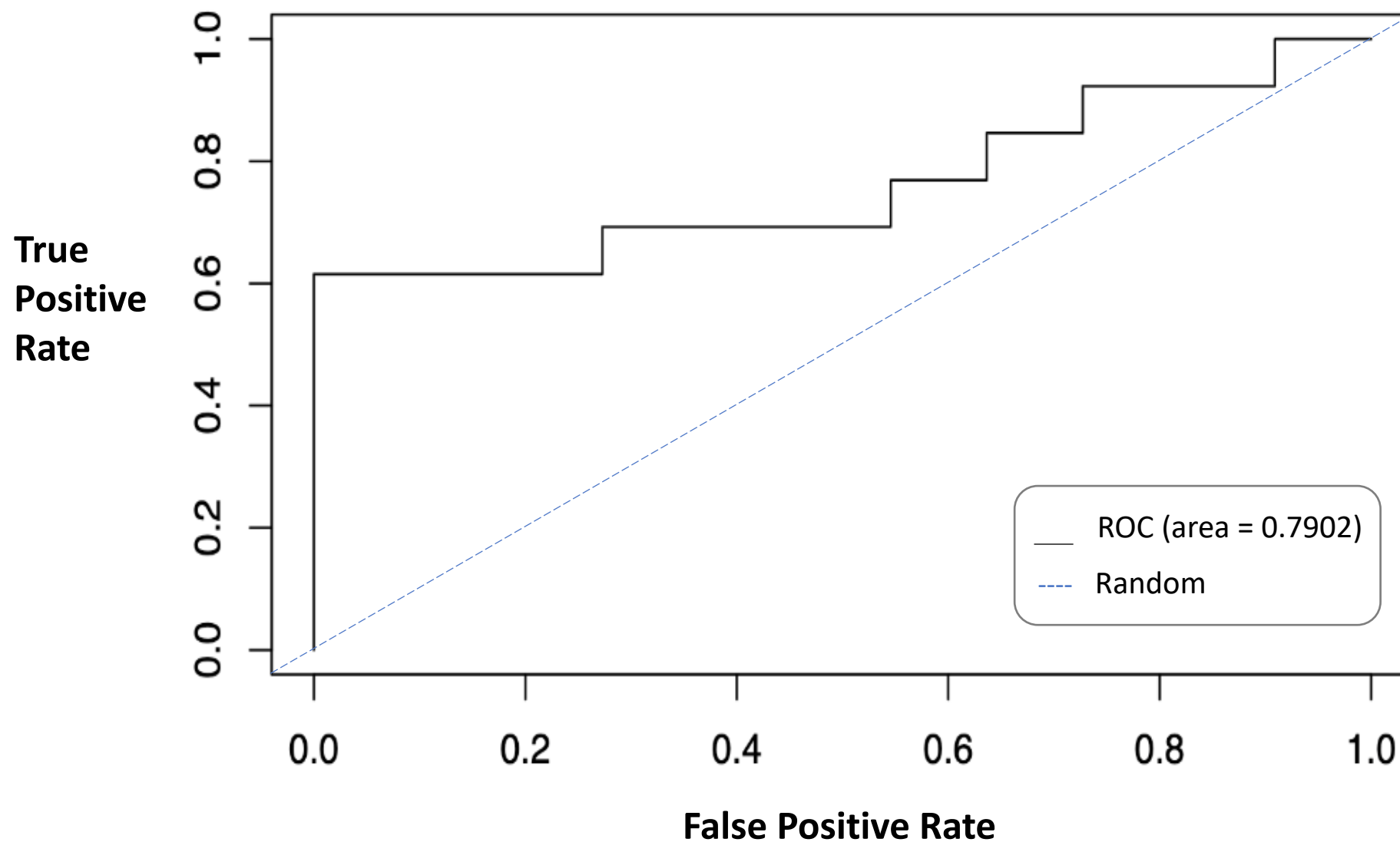

Supplement: Supplementary file 4 [file Image_3.PDF]
